# Abundance Is Not Enough: The Need for Multiple Lines of Evidence in Testing for Ecological Stability in the Fossil Record

**DOI:** 10.1371/journal.pone.0063071

**Published:** 2013-05-15

**Authors:** Judith Nagel-Myers, Gregory P. Dietl, John C. Handley, Carlton E. Brett

**Affiliations:** 1 Paleontological Research Institution, Ithaca, New York, United States of America; 2 Department of Earth and Atmospheric Sciences, Cornell University, Ithaca, New York, United States of America; 3 Department of Geology, University of Cincinnati, Cincinnati, Ohio, United States of America; University of Kent, United Kingdom

## Abstract

The fossil record is the only source of information on the long-term dynamics of species assemblages. Here we assess the degree of ecological stability of the epifaunal pterioid bivalve assemblage (EPBA), which is part of the Middle Devonian Hamilton fauna of New York—the type example of the pattern of coordinated stasis, in which long intervals of faunal persistence are terminated by turnover events induced by environmental change. Previous studies have used changes in abundance structure within specific biofacies as evidence for a lack of ecological stability of the Hamilton fauna. By comparing data on relative abundance, body size, and predation, indexed as the frequency of unsuccessful shell-crushing attacks, of the EPBA, we show that abundance structure varied through time, but body-size structure and predation pressure remained relatively stable. We suggest that the energetic set-up of the Hamilton fauna's food web was able to accommodate changes in species attributes, such as fluctuating prey abundances. Ecological redundancy in prey resources, adaptive foraging of shell-crushing predators (arising from predator behavioral or adaptive switching in prey selection in response to changing prey abundances), and allometric scaling of predator-prey interactions are discussed as potential stabilizing factors contributing to the persistence of the Hamilton fauna's EPBA. Our study underscores the value and importance of multiple lines of evidence in tests of ecological stability in the fossil record.

## Introduction

Understanding how the structure and function of ecological communities changes or remains the same through time is a topic of considerable interest [1], [2]. Much of what we know about community stability and change comes from insights gained from ecological data collected over short time intervals of up to a few decades [2]–[5]. Increasingly, however, the fossil record has proven to be a valuable ecological archive of faunal responses to disturbances over long temporal scales not available in ecological studies [2], [6], [7]. One of the most surprising insights gained from paleoecological data is that some fossil assemblages may remain relatively stable over millions of years.

The faunas of the Middle Devonian Hamilton Group of New York State provide an exemplar of this pattern. Brett and Baird [8] recognized long intervals of faunal persistence terminated by turnover events induced by environmental change (see also [9]–[11]). Nearly two decades of additional research has generally supported the original interpretation of *taxonomic stasis* in this fauna [10], [12]—in other words, large numbers of species, or closely related species groups within lineages, persist in similar facies/environments over long intervals of time.

A less well-documented pattern in the fossil record is the suggestion that faunas are also relatively stable in terms of ecology (*ecologic stasis*; *sensu* [12]). This claim has been subject to considerable discussion [13]–[17]. For instance, although guild structure appears to persist in the Hamilton fauna [10], [12], [18], several studies have challenged ecological stability expressed in terms of relative abundance data (e.g., [19]–[21]).

The unresolved issue in these cases is sample comparability. Valid comparisons of faunas of differing age, required to test for properties of ecological stability, have to be based upon the most similar biofacies; lithology alone is not sufficient. Incomplete sampling and small-scale spatial variation in faunas and environments can further obscure paleoecological data [11], [12]. Two extensive studies recently corroborated ecological stability within specific biofacies of the Hamilton fauna. For instance, Brett et al. [10] showed that guild proportions remained similar in all samples of five biofacies, ranging from relatively low diversity, dysoxic assemblages to highly diverse coral- and brachiopod-rich, shallow shelf biotas, and Ivany et al. [12] documented the constancy of the relative abundance of the diverse coral-brachiopod biofacies in 13 horizons throughout a stratigraphic interval spanning about 5 to 5.5 million years.

Here we expand upon our current understanding of the pattern of ecological stability in the fossil record. Our approach compares data on abundance structure (the standard metric used to test for ecological stability in the fossil record), body-size structure, and predation pressure in bivalve-dominated assemblages within the well-constrained stratigraphic framework of the Hamilton fauna [10], [22].

### Study system

**Figure 1:**
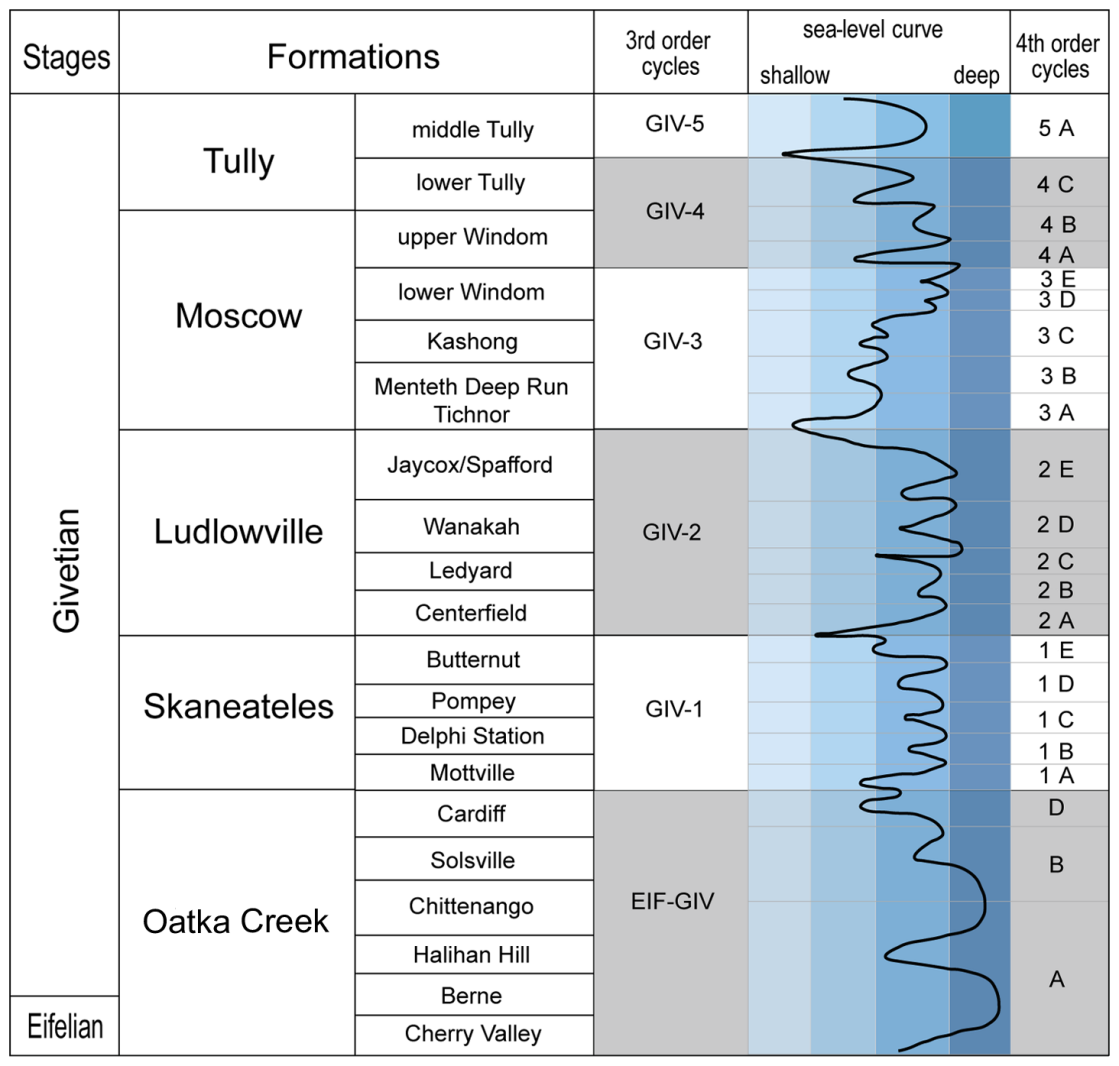
Sequence stratigraphy for the Middle Devonian of New York State.

To test for the pattern of ecological stability we focused on a particular biofacies—that of shallow, storm-affected, silty shelf bivalve-dominated assemblages—of the well-preserved Middle Devonian Hamilton fauna of New York. The Hamilton fauna comprises over 300 invertebrate species [10], [23]–[27] and occurs throughout four formations (Fig. 1): Oatka Creek, Skaneateles, Ludlowville, and Moscow, each of which is approximately a 3^rd^-order cycle of sea-level change lasting ∼1–2.0 million years [18], [27]. These units represent shallow subtidal muddy to silty shelf sediments deposited below fair weather wave base, but above storm wave base in, euphotic to dysphotic environments, ranging in water depth from about 20 to 80 meters in a warm temperate to subtropical setting [10], [23], [28]. Each of the formations is divisible into a series of 10–20 m scale, coarsening upward mudstone to siltstone members and submembers representing 4^th^-order cycles of sea-level change of ∼400 ka duration (Fig. 1; [22]). Average rates of sea-level rise during this time interval have been estimated to be around 1 to 10 mm/year based on estimates of absolute depth change of ∼40–50 m [22], [26], [28] and durations of decameter scale submembers [29]. Our study system included seven localities collected within a 2000 km^2^ geographic area, examined a duration of about 800 ka, and sampled three 4^th^-order depositional cycles—Giv-1A, Giv-1B, and Giv-1C—from the lower Givetian Skaneateles Formation (Fig. 1).

**Figure 2:**
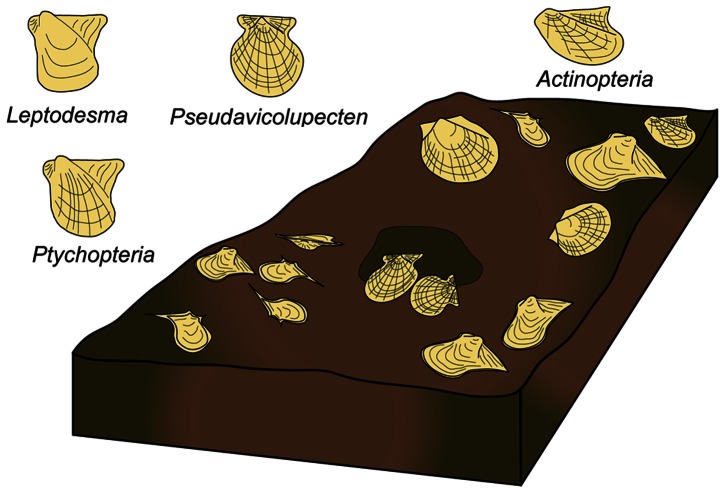
Reconstruction of epifaunal pterioid bivalve assemblage of the Hamilton fauna.

**Figure 3:**
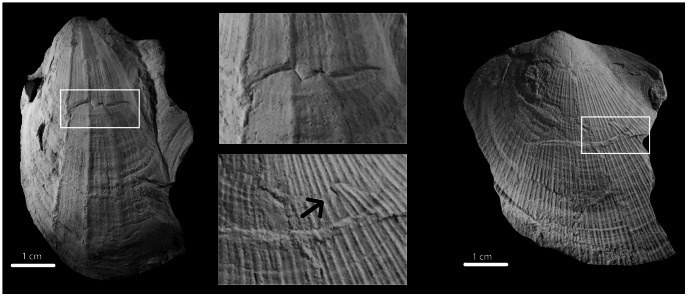
Examples of predation-induced shell repair. Left: *Ptychopteria* from Cole Hill (PRI 67471); Right: *Pseudaviculopecten* from Oran Gulf (PRI 67470); Center: close-up view of repaired shell portion of each specimen; note off-setting of ribbing patterns and high relief of repair scars.

We targeted a functional group of suspension feeding bivalves within the bivalve-dominated biofacies of the Hamilton fauna—the epifaunal pterioid bivalve assemblage (hereafter referred to as EPBA), which is composed of pterioid species that flourished in the Devonian [30]. Pterioid bivalves lived either byssally attached (*Pseudaviculopecten*) or reclining (*Ptychopteria*, *Leptodesma*, and *Actinopteria*) on soft substrates (Fig. 2). These genera reflect either single species or morphological groups of closely-related species, which comprised as much as 75% of the shallow water shelly epibenthos [31], [32]. As in many modern marine systems, this functional group would have played a key role in ecosystem function, influencing nutrient dynamics, as well as serving as food for higher trophic levels [33]. Co-occurring with this functional group of bivalves was a moderate diversity of sessile, epifaunal suspension-feeding brachiopods, bryozoans, and crinoids, endobenthic scavengers, such as trilobites and gastropods, and deposit feeders, including nuculid bivalves, with moderate bioturbation [34]. The presence of benthic, durophagous (shell-crushing) predators, such as phyllocarid crustaceans and gnathostome fishes [35], [36] is preserved in the rich trace fossil record of their attacks on bivalve prey (Fig. 3; [36]).

This predator-prey interaction forms a simple food-web module (*sensu* [37]) in which to test for ecological stability in abundance, body size, and predation in the Hamilton fauna's EPBA. This “module” approach is widely used in community ecology to help disentangle the complexity of a system by focusing on individual building blocks (e.g., specific species interactions) as a proxy for the dynamics of the whole system [38], [39].

## Results

### Abundance

The most abundant species in the EPBA was *Actinopteria* (55.6%; n = 299), followed by *Ptychopteria* (34.4%; n = 184), *Leptodesma* (5%; n = 28), and *Pseudaviculopecten* (5%; n = 27). Relative abundances of EPBA species varied from 15.3–63.8% for *Actinopteria*, 25.6–71.2% for *Ptychopteria*, 2.6–8.5% for *Leptodesma*, and 2.6–5.5% for *Pseudaviculopecten*, throughout the stratigraphic section (Table S1). Model ranking results using Akaike's Information Criterion (AIC) indicate 99.9% support for a change in relative abundance structure of the EPBA across all stratigraphic units; Bayesian Information Criterion (BIC) scores, which are less sensitive to model complexity, also indicate—with 96.7% support—that relative abundances of the EPBA differ across stratigraphic units (Table 1).

**Table 1:** Model ranking results for change in relative abundances.

Model	AIC	Akaike Wt	BIC	Bayesian Wt
[Giv-1A][Giv-1B][Giv-1C]	53.94	0.999	79.16	0.967
[Giv-1A][Giv-1B, Giv-1C]	103.93	0.000	123.33	0.000
[Giv-1A, Giv-1B][Giv-1C]	67.19	0.001	85.93	0.031
[Giv-1A, Giv-1B, Giv-1C]	109.70	0.000	122.56	0.001

Models represent cases where samples from each stratigraphic unit range from having distinct [Giv-1A][Giv-1B][Giv-1C] to the same [Giv-1A, Giv-1B, Giv-1C] relative abundance distributions. Stratigraphic unit designations (Giv-1A through Giv-1C) follow [22]. For each model fit, the Akaike Information Criterion (AIC), Akaike weight, Bayesian Information Criterion (BIC), and Bayesian weight are given. See Methods S1 for detailed explanation.

### Body size

**Figure 4:**
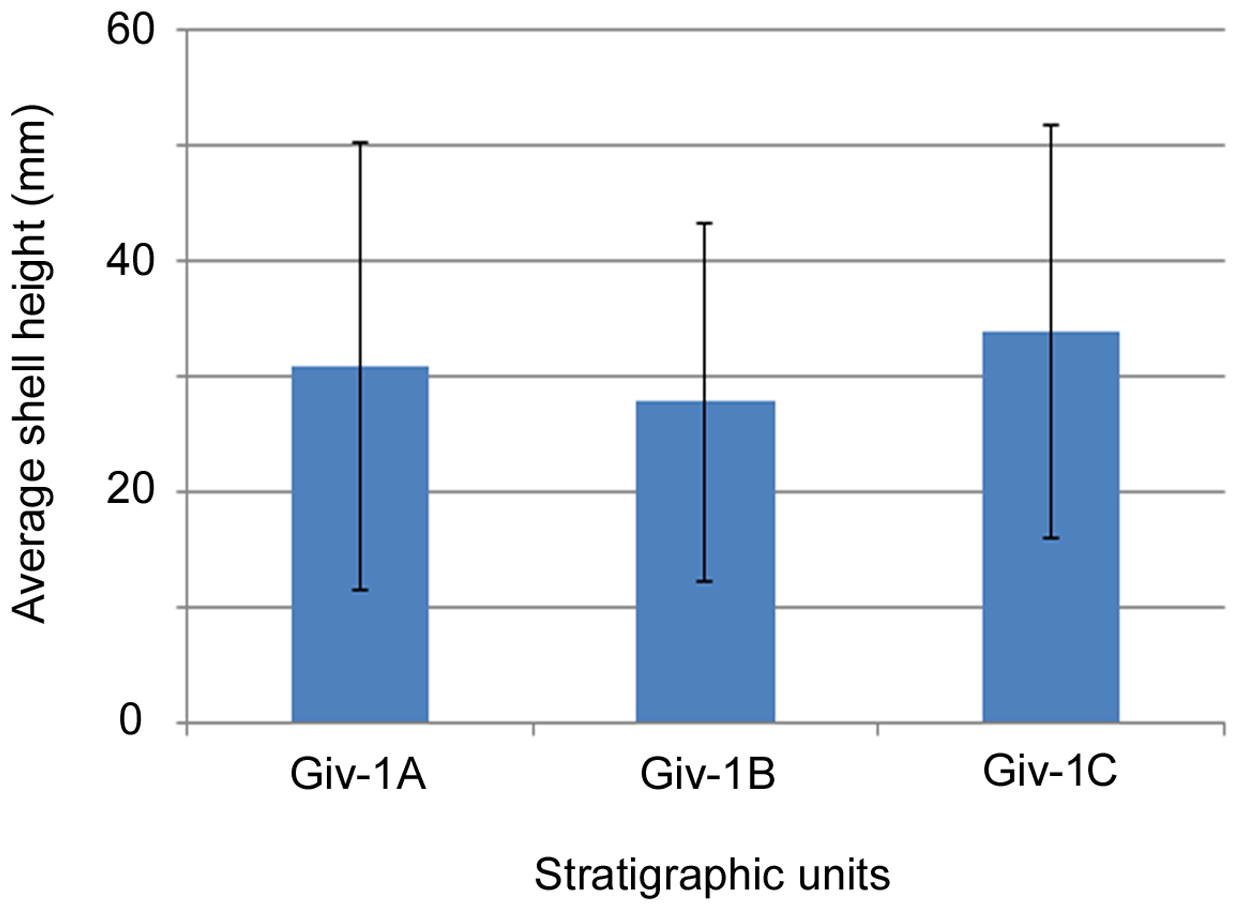
Average body-size structure of the epifaunal pterioid bivalve assemblage through time.

The average body size of the 538 bivalve specimens we examined was 28.9 mm. Average body size varied between 24.8 to 27.9 mm for *Actinopteria*, 32.5 to 35.8 mm for *Ptychopteria*, 29.4 to 40.0 mm for *Leptodesma*, and 29.7 to 34.5 mm for *Pseudaviculopecten*, throughout the stratigraphic section (Fig. 4; Table S2). Deviance and residual degrees of freedom indicate that all proposed regression models have good fits (Table 2). Model ranking results using AIC and BIC indicate no support (<0.1%) for a change in body-size structure of the EPBA across all time units (Table 2). Locality also has no effect on body size (<0.1% using both AIC and BIC; Table 2). Taxon identity, however, has a significant effect on body size (91% using AIC and 100% using BIC; Table 2), with *Ptychopteria* on average the largest (34 mm) species in the EPBA and *Actinopteria* the smallest (25 mm; Table S2). For the interaction model, there is negligible evidence that average size for each taxon changes across time units (9% and <0.1% using AIC and BIC, respectively; Table 2).

**Table 2:** Model ranking results for body size as a function of stratigraphic unit, locality, and taxon.

Model	AIC	Akaike Wt	Deviance	DF	BIC	Bayesian Wt
1	3783.988	0.000	45.472	537	3792.564	0.000
Stratigraphic unit	3756.594	0.000	42.928	535	3773.745	0.000
Locality	3743.691	0.000	41.160	530	3782.281	0.000
Taxon	3645.936	0.911	34.904	534	3667.375	1.000
Stratigraphic unit-Taxon	3650.635	0.089	34.186	526	3706.377	0.000

In the model column, ‘1’ designates a model with intercept only; otherwise the covariate is listed. Stratigraphic unit-Taxon denotes a model including unit, taxon, and unit/taxon interactions as covariates. For each model fit, the Akaike Information Criterion (AIC), Akaike weight, Deviance, degrees of freedom (DF), Bayesian Information Criterion (BIC), and Bayesian weight are given. See Methods S1 for detailed explanation.

### Predation

**Figure 5:**
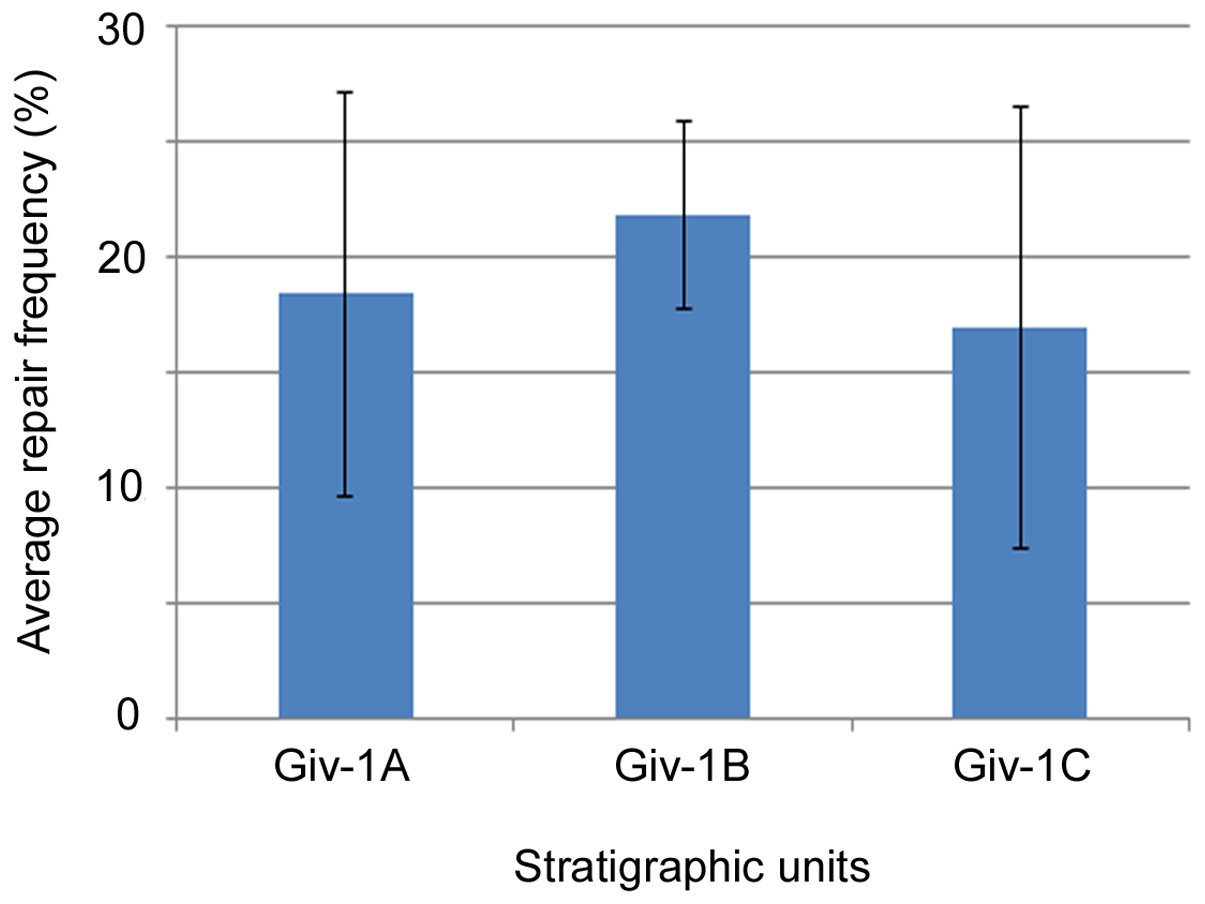
Average repair frequency of the epifaunal pterioid bivalve assemblage through time.

At least one shell-crushing repair scar was found on 112 of the 538 bivalve specimens examined in our samples (Fig. 5), with an average repair frequency (RF) of 18.3% for *Actinopteria*, 19.6% for *Ptychopteria*, 32.3% for *Pseudaviculopecten*, and 9.5% for *Leptodesma* (Fig. 5, Table S3). Repair frequency for the EPBA as a whole varied from 16.9% to 21.8% throughout the stratigraphic section (Fig. 5). All proposed logistic regression models have good fits based on upon deviance and residual degrees of freedom (Table 3). Using a threshold of 10% for significance, neither AIC nor BIC scores show appreciable support for an influence of time unit on RF (3.3% and 0.2%, respectively; Table 3). Similarly, an effect of locality and taxon on RF has little support (0.4% and <0.1% using AIC and BIC, respectively, for locality; 6.3% and <0.1% using AIC and BIC, respectively, for taxon; Table 3). There is significant support for an effect of body size on RF by both ranking methods (76.4% using AIC and 38.8% using BIC; Table 3), although the biological effect of this influence is small; the estimated coefficient of size is 0.0279 (std. err. = 0.012; p = 0.02), which suggests that for every 1 mm increase in size over the mean size there is an increase in the probability of finding a repair scar of only 0.005. The interaction model also had no support (<0.1%) by either AIC or BIC (Table 3) for RF differing across time units as a function of taxon.

**Table 3:** Model ranking results for repair frequency as a function of stratigraphic unit, locality, taxon, and body size.

Model	AIC	Akaike Wt	Deviance	DF	BIC	Bayesian Wt
1	552.410	0.141	550.41	537	556.698	0.610
Stratigraphic unit	555.323	0.033	549.323	535	568.187	0.002
Locality	560.090	0.003	546.090	531	595.967	0.000
Taxon	554.017	0.063	546.018	534	571.169	0.000
Body size	549.025	0.764	545.026	536	557.601	0.388
Stratigraphic unit-Taxon	564.741	0.000	540.740	526	616.195	0.000

In the model column, ‘1’ designates a model with intercept only; otherwise the covariate is listed. Stratigraphic unit-Taxon denotes a model including unit, taxon, and unit/taxon interactions as covariates. For each model fit, the Akaike Information Criterion (AIC), Akaike weight, Deviance, degrees of freedom (DF), Bayesian Information Criterion (BIC), and Bayesian weight are given. See Methods S1 for detailed explanation.

## Discussion

### Food-web structure and stability

Our results demonstrate that the body-size structure of, and predation pressure on, the Hamilton fauna's EPBA persisted for about 800 thousand years—despite significant fluctuations in relative abundance of individual bivalve species. Persistence of body-size structure and the interaction strength between shell-crushing predators and their bivalve prey suggests long-term stability of food-web structure. This pattern might at first seem at odds with ecological theory, which predicts that complex food webs should not persist because of their inherent instability [3], [40]–[43]. However, a growing number of studies attribute “flexibility” in food-web structure, arising from predator behavioral or adaptive switching in prey selection in response to qualitative and quantitative resource changes (e.g., changing prey abundances) in space and time, as a mechanism contributing to ecological stability (e.g., [42],[44]–[46]). Prey switching may occur passively due to predator familiarity with an encountered prey type, or actively as a “choice” made by the predator to increase fitness [47].

For this mechanism to explain the long-term ecological stability of the EPBA, Devonian predators would have had to have switched their feeding patterns, while at the same time maintaining similar predation pressures on their prey. Our data are consistent with this prediction. Although average RF throughout the study interval persisted relatively unchanged, the relative abundance and RF values of individual prey species are positively correlated (R^2^ = 0.95), supporting the prediction that predators did not have rigid feeding patterns.

In modern systems, shell-crushing predator-prey interactions also are highly size-structured, with predators often larger than their prey [48], [49]. We assume that this simple body-size relationship applies to shell-crushing predator-prey interactions in Devonian seas, given its regularity across habitat types and taxonomic groups in food webs today [50], [51]. Ecological theory predicts that species persistence is enhanced with a consistent body-size structure of predators and their prey (i.e., allometric scaling; [46], [51]), with invertebrate and vertebrate predators generally on geometric average 10 and 100 times, respectively, larger than their prey [52]. Although we do not have information on the body sizes of Devonian predators, the lack of significant change in shell-crushing predation, indexed by RF (and thus per capita effects of predators on prey), and body-size distribution of the EPBA, is indirect evidence suggesting that the predator-prey body size ratio remained high; in other words, most predators were likely to have been larger than their bivalve prey. If this general pattern did not hold in the Devonian, we would have expected change in the EPBA body-size distribution, reflecting new dynamics of the size-structured predator-prey interaction [53], [54].

Given the effects of adaptive foraging and body size on the persistence of complex food webs, a possible scenario for ecological stability of the EPBA emerges. As the size-structured predator-prey interaction between shell-crushing predators and their bivalve prey was disturbed by low-level stress (i.e., sea-level change), it is possible that this disturbance led to fluctuating selection on interaction strength in space and time and consequently food-web reconstruction (due to changes in fluctuating abundances of prey). As environmental conditions changed, different connections in the shell-crushing predator-prey module of the food web were strengthened (increasing RF values) while others were dampened (decreasing RF values). Over time this fluctuating pattern gave the shell-crushing predator-prey interaction some “flexibility”—potential connections (links) in the food web were turned off or on, while overall connectance of the module was kept low (i.e., few strong interactions; [55]) in response to sea-level changes and fluctuations in prey abundance to enhance EPBA persistence.

### Functional redundancy and ecological stability

We have shown that the relative abundance of bivalve species in the EPBA did not remain stable throughout the depositional cycles of the Hamilton fauna we sampled; however, at lower levels of resolution (e.g., presence-absence data) there is evidence that the same bivalve species were always present. This alternative conclusion is the consequence of the scale of analysis we used (i.e., the numerical resolution of the data—a problem that is not fully appreciated; [56]). Our use of relative abundance data (a high level of resolution), however, allowed us to detect more subtle changes in the structure of the EPBA, which had important effects on predator-prey interactions.

Our relative abundance data also suggest a degree of functional redundancy [57]–[62] or complementarity [63] of the prey species in the Hamilton fauna EPBA. The functional group we examined consisted of species with similar, overlapping—but not identical—niches (operating at the same scale, in the sense of how they experienced the surrounding environment): sedentary, epifaunal, suspension-feeding bivalves. We suggest that within-scale, functional overlap of bivalve taxa may also have contributed to the ecological stability of the EPBA. As the abundance of one EPBA bivalve species fluctuated (due to changes in abiotic and/or biotic environmental factors), it was compensated for—in terms of biomass and energy use—by other species. Similarly, Ivany ([13]; p. 245) suggested that redundancy “within nested sets of taxa, such that several taxa proportionately share a given ecological role and compensate for each others' short-term abundance fluctuations…” may have contributed to patterns of ecological stability in fossil assemblages. To our knowledge, our study is the first to present data supporting this speculation.

If compensatory dynamics have strong stabilizing effects, it is conceivable that changes in abundance structure of the EPBA may not have altered properties at the scale of the whole ecosystem. For instance, theoretical and empirical evidence from modern systems indicates that ecosystem-level properties, such as productivity, exhibit less variability in response to environmental change than changes in abundance of organisms [64], [65]. Conserved body-size structure (Fig. 4) in the EPBA through time is consistent with this expectation; in this way, compensatory shifts in species abundance within the EPBA may have acted as a buffer against diminished suspension-feeder biomass. We acknowledge the tentative nature of the evidence regarding this conclusion. A more rigorous test would entail collecting data on the absolute abundance of species within the EPBA, which could serve as a proxy for total biomass and energy use. We suspect, however, that such a test will not change our interpretation, given that measures of relative and absolute abundance—in organisms as diverse as trilobites and mammals—are often positively correlated (i.e., proportional to each other [66]–[68]).

### Predators and interaction modules

We assumed that the EPBA interacted with the same group of predators throughout the study interval. Similarity in shape and position of repair scars (Fig. 3; [36]) on the shells of bivalve prey supports this assumption, but is not direct evidence of taxonomic stability in the composition of the shell-crushing predator functional group. At present, only lists of possible predators are available [35], [36]. Although we do not know (and may never know) the identity of the Devonian shell-crushing predators that unsuccessfully attacked bivalve prey, our results showcase the utility of predation metrics, which estimate the strength of interaction among a few interacting species between trophic levels, in tests of long-term ecological stability in the fossil record. By focusing on a small number of interacting species—or modules of food webs [37], [39], [69]—it was possible to gain insight into ecosystem-level processes (e.g., biomass and energy use). Extending this approach to the EPBA predator-prey module throughout the remainder of the Hamilton fauna' s duration as well as other interaction modules (such as symbiosis and competition) is a fruitful avenue of future research.

### Implications for coordinated stasis

Our results have implications for understanding the pattern of coordinated stasis—long intervals of faunal persistence terminated by turnover events induced by environmental change [9]. Although coordinated stasis is a statement about observed patterns of the fossil record, and not a hypothesis about process, a number of mechanisms have been proposed to explain the pattern (see [13], for a review). For instance, ecological locking, in which “ecological interactions maintain a static adaptive landscape and prevent both the long-term establishment of exotic species…and evolutionary change of the native species…” ([70]; p. 11273) and incumbency (i.e., resistance by incumbents to invading taxa; [13]), have been widely discussed as possible intrinsic causal mechanisms to explain the pattern of coordinated stasis (e.g., [13], [70]–[73]). The extrinsic cause of habitat tracking [74]–[76], in which changes in the physical environment force organisms to migrate and to track their favored environments, is another debated [77] mechanism. Although species migrate individualistically, similar species-specific tolerance limits, among several taxa, in terms of water depth, substrate type, and other environmental parameters may give the appearance of groups of species (essentially biofacies) tracking changes in the physical environment as a unit [76]. In other words, species distributions along environmental gradients—especially those related to water depth—may remain relatively stable, but the species shift spatially as the gradients themselves shift [11].

We suggest that the energetic set-up of food webs—adaptive foraging of consumers (e.g., [42]), body-size structure of consumer-resource relationships (i.e., allometric scaling; [51]), and functional redundancy of prey species (*sensu* [57], [58], [78])—offer alternative, complementary mechanisms to explain coordinated stasis in the fossil record. We recognize that defining operational criteria for distinguishing among these alternative mechanisms will be difficult in most cases because they predict nearly the same behavior. These mechanisms also are not mutually exclusive. For instance, a low-stress disturbance (such as sea-level rise) that drives species to migrate (i.e., habitat tracking, *sensu* [10]) may result in the relative abundances of the players changing as the community is reassembled, but such change, does not necessarily overturn the ecological applecart—to use Eldredge's [79] apt description—to change the structure and function of the food web as a whole. In addition, processes may actually interact additively or synergistically, leading to even a higher level of ecological stability (e.g., interactive, stabilizing effects of body-size structure and adaptive foraging in food-webs; [46]).

Our focus on the internal dynamics of food webs shares with “ecological locking” (*sensu* [70]) an emphasis on species interactions. Ecologic locking “emphasizes the strength and structure of ecological interactions…in holding ecological relationships relatively constant so that rank abundances and guild structure do not fluctuate widely” ([13]; p. 245). This mechanism requires a tight integration of interacting species (in other words, an “intrinsic” ecological mutual dependence—the acting, reacting, and co-acting—of EPBA inhabitants, which essentially “glues” the assemblage together). Our conclusion that the EPBA food web was stable for about 800 ka, however, does not imply a “locked” interaction module of shell-crushing predators and their bivalve prey; that is, a static, highly integrated entity, in the sense of equilibrium (steady-state) notions of the term [80]. Instead, we view the stable EPBA as an open and flexible food web with variable species attributes, such as abundance and composition. The persistence of stable assemblages of interacting organisms is thus dictated by their capacity to accommodate disturbance—variation and the capacity to respond rapidly to such variation are critical to the maintenance of coordination in coordinated stasis.

### Paleoecological patterns and minimalist interpretations

Our interpretations assume that the internal dynamics of food webs can be scaled up to produce predictable patterns in the fossil record. We adopted a scale-independent view, in which patterns are similar on multiple scales of observation, although not infinitely (*sensu* [81]), because of an increasing body of evidence indicating that biological processes, such as predation, can act in similar ways across a spectrum of spatial and temporal scales (see [81]–[83] for reviews). Our data support this hypothesis. For instance, the positive correlation we found between the relative abundance of bivalve prey and RF (an index of predator selectivity)—a pattern evident at a temporal scale of hundreds of thousands of years—is consistent with modern examples of prey-switching behavior by predators occurring on vastly different temporal scales, ranging from days to thousands of years (e.g., [84], [85]). To the extent that a minimalist interpretation is adequate, the paleoecological patterns we found are thus best viewed as local changes summed over vast sweeps of space and time rather than as the result of “different rules” (i.e., scale-dependent processes [86] operating at paleontological scales).

### Decoupling of ecological patterns

Our study shows that interpretations of ecological patterns of stability in the fossil record depend on what metrics are used. We would have rejected the hypothesis of ecological stability if we only assessed patterns in relative abundance through time. Instead, a complex pattern of ecological stability emerged when other assemblage-level properties were taken into consideration. This result raises serious doubt as to whether the phenomenon can be tested meaningfully solely based upon the abundance of taxa (which has been the standard metric used to test for ecological stability in paleoecology; [15], [19]–[21]). We suggest that multiple lines of evidence are needed to increase the confidence in the signals derived from paleoecological data. Our test of ecological stability drew upon different types and sources of information, requiring the integration of multiple lines of evidence that converged (and diverged) before conclusions were reached. Our study thus underscores the critical need for multiple, comparative datasets in tests of ecological stability in the fossil record.

## Materials and Methods

### Sampling

Because it is crucial that all samples represent the same benthic association, the sampling target for our study consisted of siltstones/silty mudstones near the “caps” of coarsening upward depositional cycles or parasequences. The sampled siltstone beds were rapidly deposited and experienced within habitat time-averaging (*sensu* [87], [88]), which excludes the mixing of different depth related assemblages. Associated specimens are typically well preserved with little sign of corrasion and fragmentation; thus, the amount of time in residence on the seafloor was probably rather short (see [26], [89]). Most siltstones are not associated with evident sediment starvation, such as phosphatic nodules; however, many shells and most multi-element skeletons are disarticulated and the sediments are rather strongly bioturbated (mainly *Zoophycos*) in some cases indicating sedimentation rates low enough for fairly thorough breakdown of primary sediment structures and articulation of skeletons. Overall, time averaging on the scale of decades to a maximum of a few hundred years can be assumed for the targeted siltstone beds.

The majority of epifaunal bivalve specimens found in these siltstone beds are preserved as internal, external, or compression molds, all of which yield excellent surface detail. The morphology of these taxa is also ideal for preserving evidence of predatory attacks by durophagous (shell-crushing) predators. The pterioid taxa we studied possessed at least one valve with a simple, exterior prismatic calcite layer that made their shell highly flexible [90], [91]. This microstructural trait would have enabled Devonian pterioids to seal their shells tightly, enabling them to survive a high degree of shell damage induced by shell-crushing predators ([36]; Fig. 3), as is the case with modern bivalve groups that possess these traits [92]–[94].

At seven localities (Table 4), we target-sampled bivalve specimens in the EPBA. Ottens et al. [95] showed, if relatively common taxa are targeted, and efforts are made to collect all specimens, that targeted collecting provides results similar to those from bulk collecting. Outcrop conditions—small road cuts or stream beds—prevented the collection of replicate taxon-specific ( = targeted) samples, due to a lack of extensive and continuous exposures of the sampled siltstone beds to assess any underlying outcrop-scale patchiness [96], [97] of the EPBA. Taxon-specific sampling, however, tends to average out spatial variation within a locality [95], because the nature of the collecting process—searching a circumscribed area of an outcrop for float specimens—results in the pooling of small numbers of specimens collected from multiple sites distributed over a large sampling domain into a single sample. This sampling strategy thus has the same intrinsic advantage as combining multiple, small bulk samples to average out patchiness within a locality [97]. All specimens (Table S4) included in this study have been deposited at the Paleontological Research Institution (PRI), Ithaca, New York, USA (PRI Accessions 1552 and 1626). No permits were required for the described study, which complied with all relevant regulations.

**Table 4:** Locality list.

Locality	Latitude	Longitude	Stratigraphic unit
Lake Moraine	42°52′3.39	75°30′54.9	Giv-1C
Oran Gulf Road Cut	42°56′19.4	75°56′17.54	Giv-1B
Pompey Road Cut	42°55′12.19	75°55′36.74	Giv-1B
Pratt's Falls	42°55′55.77	75°59′45.00	Giv-1A and Giv-1C
Cole Hill Road Cut	42°50′56.53	75°25′43.19	Giv-1B
Route 92	42 57 26.38	75 53 53.28	Giv-1B
Pompey Hill	42°53′34.74	76°25′55.40	Giv-1A

### Assemblage metrics

#### Abundance

A full description of the ecological structure of fossil assemblages must include information on the abundance distribution of its members [98], [99]. It is not surprising then that the first tests of ecological stability in the Hamilton fauna assessed abundance patterns [19]–[21]. To test for stability in the abundance structure of the EPBA, we counted and identified all left valves in our samples to at least the “genus” level based on morphology. Fragments of specimens were only counted if at least one-third of the left valve was present.

#### Body size

Species assemblages are also strongly structured by body size of their members [100]. Species interactions, metabolic rate, life history and geographic distribution are all influenced by the body size of organisms [100], [101]. Therefore, information on the distribution of body sizes within an assemblage of species is a useful descriptor for a large amount of biological information reflecting the dynamics and structure of food webs [102]. To test for stability in body-size structure of the EPBA, we measured the dorso-ventral length of the left valve of all complete specimens greater than 5 mm to the nearest 0.05 mm.

#### Predation

The structure of an assemblage of species also depends on how species interact. Top-down forces (i.e., predation) have long been recognized as important community structuring mechanisms (e.g., [103]). Predators may affect prey populations directly by preying on them or indirectly by altering prey traits including behavior, morphology, or habitat use [39]. Therefore, information on the strength of interactions between predators and prey is a useful descriptor of patterns of energy use and structure of the EPBA in the Hamilton fauna. To test for stability in predation pressure, we traced the history of the interaction between epifaunal bivalves and their shell-crushing predators. We focused on this interaction because of the important role epibenthic shell-crushing predators have in structuring benthic marine communities in modern systems (e.g., [104], [105]).

We calculated an assemblage-level RF—the number of specimens with at least one repair scar on the shell [106]—in each of our EPBA samples as our proxy for predation pressure. Only repair scars identified as resulting from biotic agents were counted in our tallies. Breakage-induced shell damage that resulted from unsuccessful attacks by predators was differentiated from other non-biological taphonomic processes, such as sediment compaction, by the presence of characteristic features of damage and repair, including scar position and geometry (e.g., jagged, scalloped shape; [106]), changes in growth line banding, and loss or offsetting of minor radial surface ornamentation, if present (Fig. 3). Following Nagel-Myers et al. [36], only the left valve of specimens that preserve the outer shell layer as an external mold or compression steinkern were used in our RF analysis.

Because RF estimates are sometimes challenging to interpret in terms of predation pressure (i.e., lethal predation [106]–[109]), we standardized our data to increase confidence that comparisons were made between samples with equivalent likelihoods of accumulating repair scars [106]. We assessed potential for bias in our RF estimates by checking whether the accumulation of shell repairs was dependent on the taxon used and/or size of specimens [106], [110]–[113]. Controlling for these factors enhanced our ability to detect ecologically meaningful signals about predation pressure from RF estimates [106]. We used an “assemblage-level” approach (*sensu* [114], [115]) in calculating RF because our analysis was restricted only to an assemblage of functionally similar (suspension feeding) bivalve taxa that share a common adaptive syndrome (e.g., mantle retraction, shell microstructure, mobility etc.) and mode of life, and not the entire Hamilton bivalve fauna—which is an amalgam of heterogeneous signals that is difficult to interpret meaningfully [116], [117].

### Data analysis

Three statistical analyses were used to assess change over time. We used a multinomial model to detect changes in abundance over time, a generalized linear model to assess any effects of stratigraphic position (time unit), locality, and taxon identity on body size, and a logistic regression model to determine effects of time unit, locality, taxon identity, and body size on RF. We used model ranking techniques throughout to assess importance and significance of effects.

When presenting model ranking results, we report Akaike Information Criterion (AIC), Bayesian Information Criterion (BIC), Akaike weights, and Bayesian weights (see [118] for definitions and comparisons). There is disagreement about which criterion, AIC or BIC, is better for assessing model support. AIC is viewed as favoring more complex models when the real model is more complex than any of the candidate models. BIC is considered to be more conservative in that it requires more evidence to overturn a simple model. It also assumes that the correct model is in the set being considered and each model in the set is *a priori* equally likely. Both methods require approximations that are difficult to assess in practice (for additional paleoecological applications see [119]–[122]).

For the two regression models, in addition to model ranking results, we also report diagnostic information. Because model ranking is appropriate only for plausible models, we ensured reasonable fits by inspecting residuals and reporting model deviance with respect to the residual degrees of freedom. If the ratio of deviance to degrees of freedom is greater than two, there is evidence for defects in the model (see [123] for details and examples).

All analyses were done in ‘R’ [124]. For additional information on statistical analyses see Methods S1.

#### Abundance

We used the model-ranking methods developed by Handley et al. [125] to assess whether the abundance structure of the EPBA changed through time. To compute relative abundances through time, taxon counts for each were treated as multinomial observations drawn from an underlying ecological distribution. The optimal model of EPBA structure was selected from a set of hypotheses about those distributions, based on information-theoretic measures to assess the model's support from the data. The models considered included stasis, in which samples from each time unit share the same underlying sampling distribution, complete heterogeneity, in which each sample has a different sampling distribution, and all other ordered groupings of samples by time unit.

#### Body size

We applied a generalized linear regression model to test whether stratigraphic position (time unit), locality, or taxon identity had any effect on the body-size structure of the EPBA. Body size is a response variable with time unit, locality, and taxon serving as categorical covariates. To detect whether different taxa are changing sizes over time, we also included a model that incorporates interactions between time unit and taxon (a two-way ANOVA with interaction terms).

#### Predation

To assess whether predation pressure (indexed by RF) in the EPBA changed through time, we used logistic regression, a technique commonly used in paleoecology (e.g., [126]). Our repair data represent binary outcomes (1 = attacked, 0 = not attacked) with covariates stratigraphic position (time unit), locality, taxon identity, and body size. We tested if time unit, locality, taxon, or body size has any effect on RF. To detect whether different taxa had different RFs over time, we also included a model that incorporates interactions between taxon and time unit (a two-way ANOVA with interaction terms).

## Supporting Information

Methods S1 **Expanded explanation of statistical analyses.**
(DOCX)

Table S1 **Relative abundance by taxon and stratigraphic unit.**
(XLSX)

Table S2 **Average size of specimens by taxon and stratigraphic unit.**
(XLSX)

Table S3 **Repair frequency by taxon and stratigraphic unit.**
(XLSX)

Table S4 **Data for all analyzed scarred and unscarred specimens of the EPBA by locality and taxon.**
(XLSX)
