# Supplementary material for: Abundance Is Not Enough: The Need for Multiple Lines of Evidence in Testing for Ecological Stability in the Fossil Record
Source: PLoS One. 2013 May 15;8(5):e63071. doi: 10.1371/journal.pone.0063071 (PMC3655006; doi:10.1371/journal.pone.0063071)
Supplement: Methods S1 — Expanded explanation of statistical analyses. (DOCX) [file pone.0063071.s001.docx]

**Supplemental Methods**

***Abundance***

We employed a method introduced by Handley et al. [125] and applied to coordinated stasis in Ivany et al. [12] to test for stability in the relative abundance structure of the EPBA. Table S1 lists the counts of taxa and units. There are three multinomial observations in Table S1, A = (33, 2, 39, 2), B = (257, 22, 103, 21), and C = (9, 3, 42, 5). The statistical issue is whether these three observations arise from the same underlying sampling distribution. If so, we conclude that the taxa exhibit stasis. Details of the method are given in Handley et al. (125), but we give a brief description here.

Each unit corresponds to a multinomial sample. Mathematically, we write the probability of observing sample A as

$$P(33,2,39,2)=\frac{76!}{33!2!39!2!}p_{ac}^{33}p_{av}^{2}p_{cc}^{39}p_{gly}^{2}$$

where $p_{\mathrm{ac}}+p_{\mathrm{av}}+p_{\mathrm{cc}}+p_{\mathrm{gly}}$=1. It is possible for each unit to have a different sampling distribution, so we introduce notation to indicate that the sampling probabilities correspond to a unit: $p_{A}=(p_{ac,A}$,$p_{av,A},p_{cc,A},p_{gly,A})$, $p_{B}=(p_{ac,B}$,$p_{av,B},p_{cc,B},p_{gly,B})$, and $p_{C}=(p_{ac,C}$,$p_{av,C},p_{cc,C},p_{gly,C})$. There are four possible models, which we write as, [$p_{A}]{[p}_{B}]{[p}_{C}]$,${[p}_{A}]{[p}_{B}=p_{C} ]$, $[p_{A}=p_{B}]{[p}_{C}]$, ${[p}_{A}=p_{B}=p_{C}]$, and refer to as models 1 through 4. Model 1 represents the case where all sampling distributions are different. This corresponds to the ecological setting where the relative abundances of each taxon are different for each unit. Model 4 is stasis: Each sample is drawn from the same underlying probability distribution because the relative abundances are the same. The other two models represent cases where there are change points in the underlying samples. That is, two samples from adjacent times are drawn from the same underlying relative abundances, but the third is different.

Each model can be fitted to data, maximum likelihood estimates of parameters computed, and an information theoretic score calculated. The model with the lowest score is deemed the best. In general, if $x=(x_{1},\ldots,x_{n})$ represents a set of data and $f(x;\theta)$ represents its probability density function, where $\theta$ is a vector parameter with *K* components, then there are two common information theoretic measures of relative model aptness for the data. The first is Akaike’s Information Criterion, or AIC,

$$AIC=-2\text{log}f\left( x;\theta_{mle} \right)+2K$$

where $\theta_{mle}$ is the maximum likelihood estimate of $\theta$. Intuitively, AIC measures a tradeoff between fitting the data well and the number of parameters in the model to fit the data. The other information theoretic measure is Bayesian Information Criterion, or BIC. It has a similar structure,

$BIC=-2\text{log}f\left( x;\theta_{mle} \right)+K\text{log }n$.

BIC again trades off fitting the data with model complexity, but weights the complexity with the number *n* of data points. In the lore of model ranking, AIC is believed to favor more complex models than BIC (referred to as over fitting the data). Often both are presented.

The maximized likelihood for model 1 (which has 9 parameters) is

$$f_{1} = \frac{76!}{33!2!39!2!}\left( \frac{33}{76} \right)^{33}\left( \frac{2}{76} \right)^{2}\left( \frac{39}{76} \right)^{39}\left( \frac{2}{76} \right)^{2}\frac{197!}{97!14!71!15!}\left( \frac{97}{197} \right)^{97}\left( \frac{14}{197} \right)^{14}\left( \frac{71}{197} \right)^{71}\left( \frac{15}{197} \right)^{15}$$

$$\times\frac{59!}{9!3!42!5!}\left( \frac{9}{59} \right)^{9}\left( \frac{3}{59} \right)^{3}\left( \frac{42}{59} \right)^{42}\left( \frac{5}{59} \right)^{5}.$$

The maximized likelihood for model 4 (which has 3 parameters) is

$$f_{4} = \frac{538!}{299!27!184!28!}\left( \frac{299}{538} \right)^{299}\left( \frac{27}{538} \right)^{27}\left( \frac{184}{538} \right)^{184}\left( \frac{28}{538} \right)^{28}.$$

Let the corresponding AIC scores be denoted, ${AIC}_{1},{AIC}_{2},{AIC}_{3},{AIC}_{4} .$ The model with the lowest information score is deemed the best “explanation” of the data given the models in the set. One can determine the relative fitness of the models by using weights. For AIC, Akaike weights are calculated using the difference from the minimum score,$\Delta_{i}={AIC}_{i}-\min_{j=1,\ldots,4} {AIC}_{i}$. Akaike weights are then,

$$w_{i}=\frac{exp(-\frac{\Delta_{i}}{2})}{\sum_{j=1}^{4} exp(-\frac{\Delta_{j}}{2})} .$$

Akaike weights sum to one and express the relative probability the model is true given that the true model is among the set of candidate models. In practice, Akaike weights tell the relative support for each model. For BIC, one makes a similar calculation using $\Delta_{i}={BIC}_{i}-\min_{j=1,\ldots,4} {BIC}_{i}$, but with a different interpretation. These weights are called posterior model probabilities (assuming each model has the same prior probability of being correct; [118]). For simplicity, we call these Bayesian weights. We interpret them as the approximate probability of each model being true, given the data.

From a practitioner’s point of view, Akaike weights and Bayesian weights represent two complementary views of weighting model complexity. Clearly, no model that weights well in either paradigm should be considered.

***Body size***

We applied a generalized linear regression model to test whether stratigraphic position (as represented by time unit), locality, or taxon identity had any effect on the body-size structure of the EPBA. In this case, the response is real-valued and all covariates are categorical (time unit, locality, and taxon). We obtain a better fit to the data by assuming the response distribution is gamma (instead of normal as in ordinary least squares linear regression). We fit models that include a single covariate as well as a two-way ANOVA with an interaction term for taxon and time unit to test for changes in size within taxa through time. Because this latter model may be unfamiliar, we provide some detail. (Single covariate models with categorical variables are special cases of ANOVA with only one term.)

For each level *a* and *b* of taxon and time unit we assume that size $S_{ab}$ has a gamma distribution with expected value, $E\left[ S_{ab} \right]=\mu_{ab}$. The data are comprised of observations for each specimen *i* of the form $(s_{i},a_{i},b_{i})$ where $s_{i}$is measured size, $a_{i}$ is taxon, and $b_{i}$ is time unit. In the parlance of generalized linear models, a link function relates expected values with covariates, often in a nonlinear way. The natural link function for the gamma generalized linear model, is *inverse*, which links expected value of sizes under each combination of levels with the linear model of covariates. For a two-way ANOVA with interaction terms, this is

$$1/\mu_{ab}=\alpha_{0}+\alpha_{a_{i}}+\alpha_{b_{i}}+\alpha_{a_{i},b_{i}}$$

where $\alpha_{a_{i}}$ = the effect of the observed taxon (a categorical value taking one of four levels, AC, AV, PT, or LE), $\alpha_{b_{i}}$ = the effect of the observed time unit (a categorical value taking on one of three values, Giv-1A, Giv-1B, or Giv-1C), and $\alpha_{a_{i},b_{i}}$ = the effect of the observed combination of taxon and time unit (one of 12 levels). Models are fit to the data using maximum likelihood, checked for lack of fit, and then ranked using AIC and BIC.

Our criteria for model fit include inspection of residuals and reporting of model deviance. Deviance, which is analogous to squared error for linear models and defined as

$$D=-2\log f\left( x;\theta_{mle} \right),$$

is used to assess model fit. A poorly fitting model would have a deviance significantly larger than the degrees of freedom. Zuur et al. [123] describes applications of generalized linear models to problems of ecology.

***Predation***

We used logistic regression to assess whether repair frequency was affected by stratigraphic position (as represented by time unit), locality, taxon identity, or body size. Data are binary outcomes (1 = attacked, 0 = not attacked) with covariates time unit, locality, taxon, and body size. We also include a two-way ANOVA with interactions to test for changes in repair frequency within taxa through time. Logistic regression is a special case of generalized linear models where the response is Bernoulli and the link function is logistic—we use the same assessment methods as in the Body Size analysis. The two-way ANOVA with interactions model and the model with single categorical variable also follow the descriptions given in the Body Size section above. What is different here is that we use the continuous value size as a covariate. This is also the model we find most supported by the data and the one we describe next.

Logistic regression models binary responses by a logit transformation of occurrence probabilities. Let $Y_{i}=1$ denote that specimen *i* shows a repair scar. Specimen *i* also has measured covariates including $s_{i}$ = size. The logistic regression equation is,

$$\text{log}\left( \frac{p_{i}}{1-p_{i}} \right)=\beta_{0}+\beta_{1} s_{i}.$$

The estimated probability *p* of a repair scar in terms of size *s* and coefficient estimates${(\hat{\beta}}_{0},\hat{\beta}_{1})$ is,

$p=1/(1+exp\left( -\left( \hat{\beta}_{0}+\hat{\beta}_{1} s \right) \right)$.

See Payne et al. [126] for an application of logistic regression in paleoecology.
